# Sir Duncombe Mann's Record of Work

**Published:** 1915-01-09

**Authors:** 


### HONOUR FOR A POOR-LAW OFFICIAL.

### Sir Duncombe Mann's Record of Work.

The well-deserved honour of knighthood conferred upon Sir Thomas Duncombe Mann will meet with the hearty approval of all those who know him, whether in his private or official capacity. It is the recognition of many years of strenuous and able work in the public service, and of the increasing importance of Poor-Law and Public Health administration in our present-day system of local government.

Sir Thomas was appointed clerk to the Metropolitan Asylums Board in 1891. The immense development which has taken place since that time in the scope of the Board's work required that its chief officer should possess exceptional administrative and intellectual ability, a great capacity for hard work, and an ardent love of the work itself. Fortunately all these qualities were found in the person of Sir Thomas Duncombe Mann, and the high position held by the Board to-day owes not a little to the sagacity with which he has guided its councils. Soon after his appointment the Public Health Act of 1891 abolished the power of the Guardians to recover from the patient or his relatives the expenses of his maintenance in the Board's fever hospitals. The result of this enactment, combined with the increased facilities provided for the removal of infectious cases, more than trebled the number of admissions to the fever hospitals in the course of the following ten years. A further extension of work occurred in 1897, when the Local Government Board issued an Order entrusting to the Managers the duty of providing accommodation for children suffering from contagious diseases of the eye, skin, or scalp; for children requiring convalescent or seaside treatment, for mentally and physically defective children, and for juvenile prisoners on remand. More recently the casual wards in London and the whole of the duties connected with the control of vagrancy in the Metropolis have been transferred from the various Boards of Guardians to the Managers. The striking improvement resulting from this change of administration has received notice in our columns on several occasions.

Finally, we may note, during the last few months the Board has taken an important part in the provision of hospital accommodation for tubercular patients, and this part of the work will undoubtedly assume greater importance in the near future. Coincident with this enlargement of the Board's work there has been a steady improvement in administration and in the quality of the work performed. The clinical instruction of medical students in the fever hospitals, inaugurated in 1889, has been organised and improved, and is now a compulsory part of the medical student's curriculum. Experts have been appointed not only for the various branches of medical work, but also in connection with the supply of stores to the institutions. Provision has been made for the manufacture of the diphtheria antitoxin used in the Board's hospitals. Research work has been encouraged amongst the medical officers, and the post of research pathologist has been created. The ambulance system has been extended to the conveyance of persons suffering from non-infectious diseases, and considerably improved by the substitution of motor for horse traction, and in other ways. The work of the statistical department has been vastly increased. Instead of each institution being controlled by independent committees three central committees were formed in 1898 to deal with the three main branches of the Managers' work—the children, the insane, and the fever patients. Each institution is now controlled by a sub-committee of one of these central committees. Uniformity in the administration of all the institutions has resulted, and a general levelling up to a high standard of efficiency. Central stores for the reception of manufactured non-perishable goods have been established, and in the case of some articles direct purchase in the open market has been introduced instead of inviting tenders for their supply. As a consequence there has been a great saving in cost and an improvement in the quality of the goods obtained.

This brief summary will give some idea of the great changes that have taken place in the scope and character of the work during Sir Thomas Duncombe Mann's years of office. The important part he has taken in it is known to all who are familiar with the municipal life of London, and it is only the bare truth to say that much of the success of the Board's policy is due to the energy, ability, and personality of its chief officer.

